# Supplementary material for: Pre-Operative Decitabine in Colon Cancer Patients: Analyses on WNT Target Methylation and Expression
Source: Cancers (Basel). 2021 May 13;13(10):2357. doi: 10.3390/cancers13102357 (PMC8153633; doi:10.3390/cancers13102357)
Supplement: Supplementary file 1 [file cancers-13-02357-s001.zip › Table S1 RIN values.pdf]

Patient sample	RIN value
Patient 1, biopsy 1	7
Patient 1, biopsy 2	5.90
Patient 1, resection specimen 1	6.30
Patient 1, resection specimen 2	7.60
Patient 2, biopsy 1	8.70
Patient 2, biopsy 2	8.30
Patient 2, resection specimen 1	7.50
Patient 2, resection specimen 2	8.20
Patient 3, biopsy 1	8.20
Patient 3, biopsy 2	7.80
Patient 3, resection specimen 1	n/a
Patient 3, resection specimen 2	n/a
Patient 4, biopsy 1	7.20
Patient 4, biopsy 2	7.70
Patient 4, resection specimen 1	7.90
Patient 4, resection specimen 2	8.80
Patient 5, biopsy 1	6.90
Patient 5, biopsy 2	7.10
Patient 5, resection specimen 1	7.70
Patient 5, resection specimen 2	8.80
