# Supplementary material for: Pre-Operative Decitabine in Colon Cancer Patients: Analyses on WNT Target Methylation and Expression
Source: Cancers (Basel). 2021 May 13;13(10):2357. doi: 10.3390/cancers13102357 (PMC8153633; doi:10.3390/cancers13102357)
Supplement: Supplementary file 1 [file cancers-13-02357-s001.zip › Table S2.pdf]

Table S2: Sequencing primers used for pyrosequencing

Gene	forward	reverse	sequencing
APCDD1	AGGTTTTAGAGTAGGATTGGAAATGT	Biotin-ACCCCCTCTCCCAAAACTA	AGTAGGATTGGAAATGTT
AXIN2	GGGAGTAGTTTTTTTGGAGTTGAT	Biotin-AACACAACCTTCCAAAAACC	TTTTTGGAGTTGATGGTA
DKK1	Biotin-GGTTTTGTTGTTTTTTTTTTAAGG	CACTTTACAAACCTAAATCCC	ACAAAAAACACAAAACTCT
LINE1	TTGAGTTAGGTGTGGGATATAGTT	Biotin-CAAAAAATCAAAAAATTCCCTTTC	ATTAGGGTGGGAGTGA
