# Supplementary material for: Pre-Operative Decitabine in Colon Cancer Patients: Analyses on WNT Target Methylation and Expression
Source: Cancers (Basel). 2021 May 13;13(10):2357. doi: 10.3390/cancers13102357 (PMC8153633; doi:10.3390/cancers13102357)
Supplement: Supplementary file 1 [file cancers-13-02357-s001.zip › Table S3.pdf]

Table S3: Primers used for rt-PCR

Gene	Forward	Reverse
APCDD1	CATCCAGACAGCAGGTCTCA	GGGCCTGACCTTACTTCACA
AXIN2	CTCCTTATCGTGTGGGCAGT	CTTCATCCTCTCGGATCTGC
DDX58	CCAGCATTACTAGTCAGAAGGAA	CACAGTGCAATCTTGTCATCC
DKK1	GCCCCGGGAATTACTGCAAAAATG	CCGGAGACAAACAGAACCTTCTTGTC
ERVL	ATATCCTGCCTGGATGGGGT	GAGCTTCTTAGTCCTCCTGTGT
GAPDH	AATCCCATCACCATCTTCCA	TGGACTCCACGACGTACTCA
LINE1	TTACCCCAGGGTGAATCACGA	GGACATTGCGATTTCCATCTCT
OASL	GCAGAAATTTCCAGGACCAC	CCCATCACGGTCACCATTG
