# Supplementary material for: Pre-Operative Decitabine in Colon Cancer Patients: Analyses on WNT Target Methylation and Expression
Source: Cancers (Basel). 2021 May 13;13(10):2357. doi: 10.3390/cancers13102357 (PMC8153633; doi:10.3390/cancers13102357)
Supplement: Supplementary file 1 [file cancers-13-02357-s001.zip › Table S4.pdf]

Table S4: Primers used for mutation analyses

Gene	Forward	Reverse
KRAS	GTGTGACATGTTCTAATATAGTCA	GAATGGTCCTGCACCAGTAA
P53	GCTTTCCACGACGGTGACA	TTGTTGAGGGCAGGGGAGTA
	TGTCATCTTCTGTCCCTTCCC	GATGGTGGTACAGTCAGAGC
	TTGCGTGTGGAGTATTTGGA	CAGTGGTTTCTTCTTTGGCTG
	AAGAAAGGGGAGCCTCACCA	GCAAGCAAGGGTTCAAAGACC
BRAF wt	AGGTGATTTTGGTCTAGCTACAGT	TAGTAACTCAGCAGCATCTCAGGGC
BRAF mut	AGGTGATTTTGGTCTAGCTACAGA	TAGTAACTCAGCAGCATCTCAGGGC
