# Supplementary material for: Pre-Operative Decitabine in Colon Cancer Patients: Analyses on WNT Target Methylation and Expression
Source: Cancers (Basel). 2021 May 13;13(10):2357. doi: 10.3390/cancers13102357 (PMC8153633; doi:10.3390/cancers13102357)
Supplement: Supplementary file 1 [file cancers-13-02357-s001.zip › Table S6 haemological toxicity.pdf]

	**Hb (mmol/L)**		**Leucocytes (x 10^9/L)**		**Thrombocytes (x 10^9/L)**	
	**Pre**	**Post**	**Pre**	**Post**	**Pre**	**Post**
1	6.8	6.8	10	na	361	na
2	10.0	9.3	6.2	5.1	222	155
3	8.7	9.0	10.3	7.5	218	194
4	9.0	8.5	10.0	6.3	221	144
5	8.5	7.9	9.2	6.9	281	245
6	8.9	8.1	9.1	8.3	258	215
7	9.3	8.5	9.4	6.3	283	207
8	7.1	6.7	8.4	7.6	457	358
9	9.4	7.5	6.0	4.6	300	197
10	10.1	8.2*	7.0	5.4*	187	130*

* Samples were taken the day after surgery
na = not available
