# Supplementary figures and images for: Pre-Operative Decitabine in Colon Cancer Patients: Analyses on WNT Target Methylation and Expression
Source: Cancers (Basel). 2021 May 13;13(10):2357. doi: 10.3390/cancers13102357 (PMC8153633; doi:10.3390/cancers13102357)

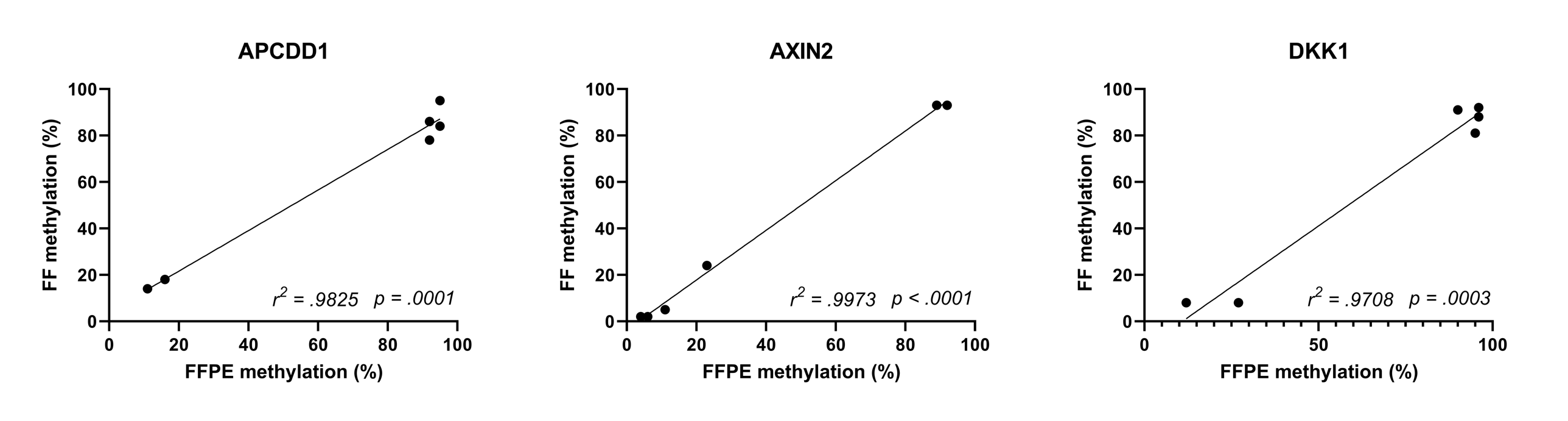

Supplement: Supplementary file 1 [file cancers-13-02357-s001.zip › Figure S1.jpg]
